# Cementoblastoma Relating to Right Mandibular Second Primary Molar

**DOI:** 10.1155/2016/2319890

**Published:** 2016-09-22

**Authors:** Sivakumar Nuvvula, Swapna Manepalli, Abinash Mohapatra, Sreekanth Kumar Mallineni

**Affiliations:** Department of Paedodontics and Preventive Dentistry, Narayana Dental College, Nellore, Andhra Pradesh 524003, India

## Abstract

Cementoblastoma is a benign lesion of the odontogenic ectomesenchymal origin. It rarely occurs in primary dentition. This report describes a case of a cementoblastoma relating to the right mandibular second primary molar in a 7-year-old girl. Her panoramic radiograph revealed a well-defined radiopaque lesion with a radiolucent border extending from the distal surface of the mandibular right first primary molar to the distal surface of mandibular second primary molar. The tumor was attached to the mesial root of primary second molar and was excised along with the teeth involved and sent for histopathological evaluation, which showed irregular trabeculae of mineralized tissue interspersed with fibrovascular connective tissue, trabeculae of mineralized tissue with prominent reversal lines, and peripheral rimming of the mineralized tissue with blast cells. On a six-month follow-up, there has been no recurrence of the lesion.

## 1. Introduction

Odontogenic tumors are the ones arising from the tissues of the odontogenic apparatus. These tumors are derived from ectodermal tissue (epithelial tumors) or from mesodermal tissue (connective tissue tumors) or are composed of both components (mixed or composite odontogenic tumors). Cementoblastomas are benign lesions of the odontogenic ectomesenchyme that rarely occur in the primary dentition. Cementoblastoma is a true neoplasm of cementum or cementum-like tissue formed on the tooth root by cementoblasts [1]. Occurrence of these lesions is more common in young patients, with about 50% of them arising under the age of 20 years. Most of the cementoblastomas are closely allied to and partly surround a root or roots of a single erupted permanent tooth [2]. The present case report describes a true cementoblastoma with relation to the right second primary mandibular molar in a 7-year-old child along with the radiographic and histological findings of the lesion in detail.

## 2. Case Report

**Figure 1:**
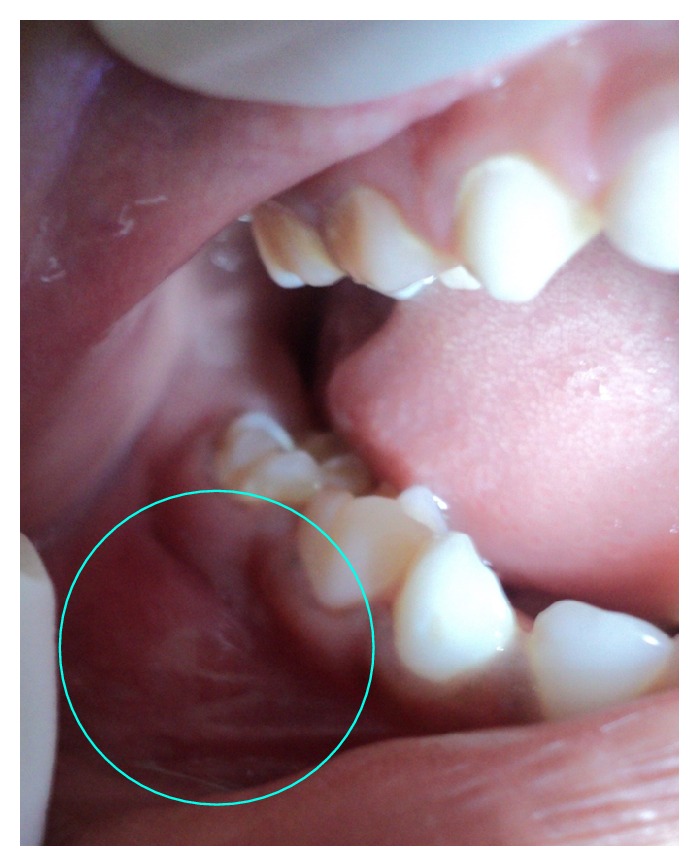
Intraoral appearance of the swelling of the lesion in relation to 84 and 85.

**Figure 2:**
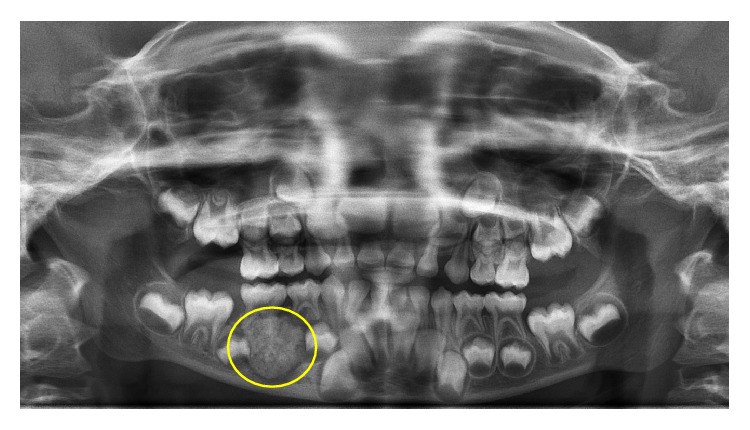
Preoperative panoramic radiograph with radiopacity surrounded by a radiolucent border associated with mesial root of lower right second primary molar.

A healthy 7-year-old girl reported to the Department of Paedodontics and Preventive Dentistry (Narayana Dental College and Hospital, Nellore, Andhra Pradesh, India) complaining of swelling on the right side posterior region of the mandibular arch. The swelling was firm and tender on palpation, which was first noticed 2 months back and increasing in size. The child presented with primary dentition and oral hygiene was adequate. Positive response was evident in both primary right mandibular molars to vitality test and the teeth were structurally sound (Figure 1). On radiographic examination OPG (orthopantomogram) showed a well-described calcified mass surrounded by a radiolucent halo measuring around 2.8 × 2.1 cm. The internal structure had a mixed radiolucent-radiopaque aspect with a wheel spoke pattern. Moreover, the lesion was associated with the roots of right mandibular second primary molar (Figure 2). The appearance of the lesion on OPG was suggestive of cementoblastoma. Differential diagnosis of this lesion included osteoblastoma, odontoma, periapical cemental dysplasia, condensing osteitis, and hypercementosis that were discussed below:

1. Cementoblastoma and osteoblastoma are very similar histologically; however, the cementoblastoma has a strict association with the root, whilst osteoblastoma arises in the medullary cavity of a wide range of bones.
2. Odontome is usually not linked to the root and has also a heterogeneous radiopacity showing the presence of multiple dental tissues [3].
3. Periapical cemental dysplasia is a smaller lesion and tends to mature to create a mixed radiographic appearance of radiolucent and radiopaque. In the later stage, the lesion shows a circumscribed dense calcification surrounded by a narrow radiolucent rim but the periodontal ligament is intact and fusion to the tooth is not present.
4. Condensing osteitis is a circumscribed radiopaque mass of sclerotic bone surrounding and extending below the apex of the root but does not show the well-defined peripheral radiolucent rim typical of the cementoblastoma and also periodontal ligament space is widened and this is an important feature in distinguishing it from the cementoblastoma [1, 4].
5. Hypercementosis radiographically demonstrates a thickening or blunting of the root. The enlarged root is surrounded by radiolucent periodontal ligament space and adjacent intact lamina dura. On rare occasions, the enlargement may be significant enough to mimic a cementoblastoma. However, cementoblastoma is distinguished on the basis of associated pain, cortical expansion, and continued enlargement. Hypercementosis is a small lesion without pain or swelling and involves nearly the entire root area, although in some instances the cementum formation is focal, usually occurring at the apex of a tooth [1, 4, 5].

**Figure 3:**
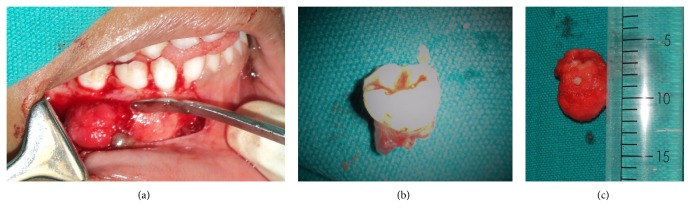
Firm and well demarcated swelling after elevation of mucoperiosteal flap (a), tooth involved (b), and the excised tissue (c).

**Figure 4:**
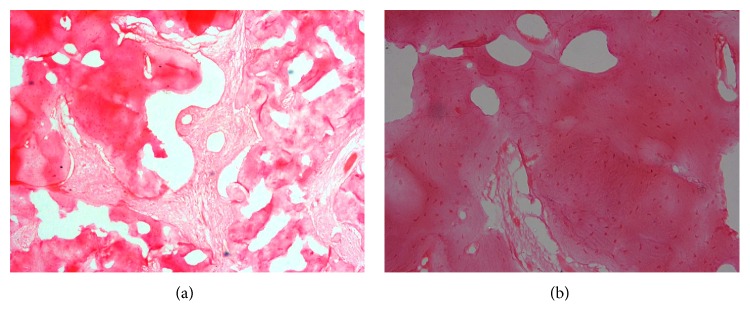
Showing irregular trabeculae of mineralized tissue interspread with fibrovascular connective tissue under 10x magnification (a) and 40x magnifications (b).

**Figure 5:**
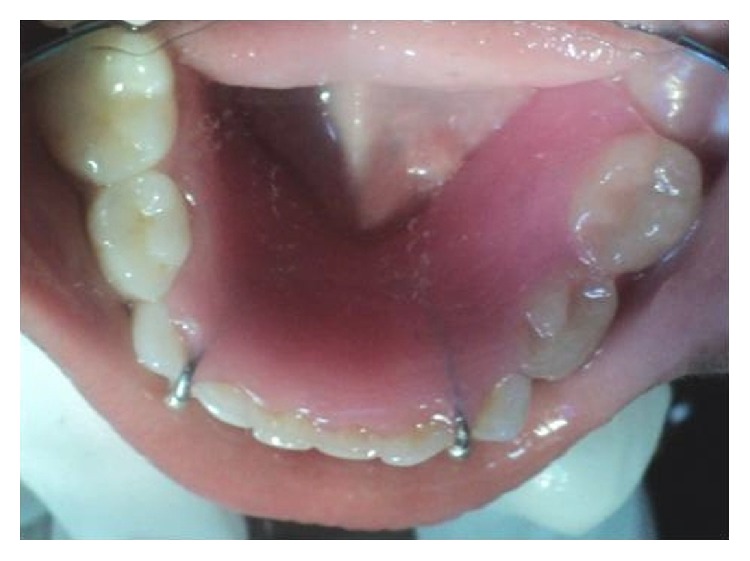
Postoperative intraoral view with a removable functional space maintainer.

**Figure 6:**
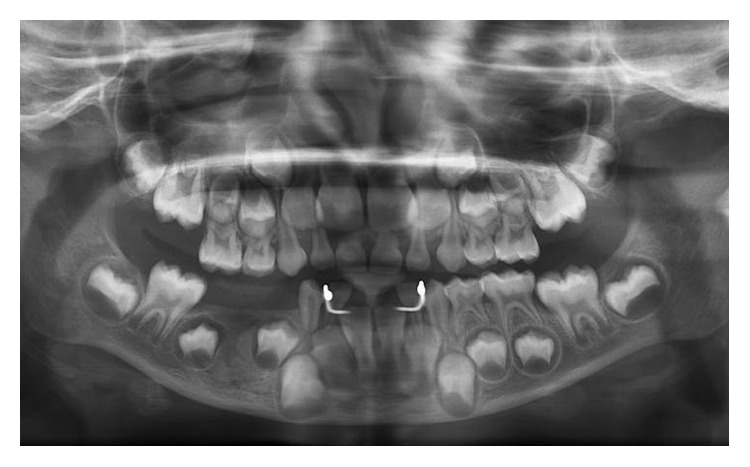
Postoperative panoramic radiograph after 6 months of follow-up with a space maintainer and no evidence of recurrence.

The decision was made for an excisional biopsy and for histopathological evaluation to confirm the final diagnosis. The excisional tissue was well demarcated and easily excised measuring 3.0 × 2.2 × 3.0 cm, almost shelling out with the attached second primary molar (Figure 3). The histopathological evaluation was performed in Department of Oral and Maxillofacial Pathology, Narayana Dental College and Hospital, Nellore. After analysing, the lesion portrayed irregular trabeculae of mineralized tissue interspersed with fibrovascular connective tissue (Figure 4(a)), trabeculae of mineralized tissue with prominent reversal lines (Figure 4(b)), and peripheral rimming of the mineralized tissue with blast cells. Postsurgical follow-up after 1 week as well as at 3 months interval was carried out. A removable functional space maintainer was fabricated and inserted in order to maintain the space and also to increase masticatory efficiency of the patient, as the permanent first molars 36 and 46 had not erupted (Figure 5). After 6 months of follow-up, the panoramic radiograph revealed no recurrence of the lesion (Figure 6). Further treatment was planned for a fixed nonfunctional lingual arch appliance in order to maintain the space for the eruption of mandibular right premolars, after the eruption of the mandibular first permanent molars and the incisors.

## 3. Discussion

Cementoblastoma is a rare lesion that represents <1% of the odontogenic tumors. The most involved area is the mandible (50% molar and premolar area) and is never associated with the anterior teeth [6]. Comprehensive search of published data retrieved a total number of 14 cases that have been reported in relation to primary teeth including the present case. Reported cases of cementoblastoma in association with primary teeth (Table 1) are interpreted, respectively [2, 3, 7–17]. Females (78.5%) are more commonly reported with cementoblastoma than males (21.5%). This pathology is more common in mandibular arch (93%) than the maxillary arch (7%). Cementoblastoma is commonly seen on right side (71.5%) of mandibular arch, followed by left side of the mandibular arch (21.5%) and right side of the maxillary molar region (7%), the most common tooth affected being right mandibular second molar (71%).

The cementoblastoma is a rare neoplasm derived from odontogenic ectomesenchyme of cementoblast that forms cementum layer on the roots of a tooth. The primary distinguishing feature for cementoblastoma is its connection to the root of the offending tooth [5]. The histological features of cementoblastoma include cementum-like tissue with numerous reversal lines and, between these mineralized and trabecular hard tissues, fibrovascular tissue with cementoblast-like cells is present along with multinucleated giant cells [5]. The prevalence of cementoblastomas in the general population has been reported to be 1.79% [7]. Of all the reported odontogenic tumors their prevalence has been reported to vary from 0.69% to 8% [18, 19].

The radiographic appearance of a cementoblastoma is a well-defined radiopacity surrounded by a radiolucent zone. Cementoblastoma occurs most often in the mandible, attached to the roots of premolar or molar teeth [20, 21]. Clinically, it involves the expansion of bone, swelling, and pain. It does not recur if the tumor/lesion is completely removed [5]. Incomplete excision and removal should be avoided, as a recurrence rate, as high as 37.1%, has been reported [22]. The male-to-female ratio for the prevalence of cementoblastoma has been reported to be 2.1 : 1, with a mean age of 20.7 years [3, 23]. Cundiff [23] suggested that radiographs should be taken at yearly intervals postoperatively that help in differential diagnosis and some criteria to distinguish the cementoblastoma from similar-appearing lesions. Cementoblastomas associated with primary teeth are extremely rare and only thirteen cases have been reported before this case and there was no observed recurrence in those cases where follow-up was carried out [2, 3, 7–17]. While the prognosis of the cementoblastoma is excellent, the recurrence is closely associated with the surgical removal of the tumor* en masse*. Radiological and clinical follow-up is therefore a mandatory investigation in these cases for better patient management. The present case met all the clinical, radiographic, histological, and surgical criteria that are suggestive of a cementoblastoma.

## 4. Conclusion

Despite being a rare condition in the primary dentition, it is essential to increase the awareness of this type of lesions among general and paediatric dentists as well as acquaintance with the clinical, radiographic, and histological findings and treatment options which can be rendered for better patient compliance.

## Figures and Tables

**Table 1:** Reported cases of cementoblastoma associated with primary teeth.

Author	Year	Age (Y)/sex	Involved teeth
Chaput and Marc [7]	1965	10/F	85 and 44
Vilasco et al. [8]	1969	8/F	85
Zachariades et al. [9]	1985	7/F	84, 85, 46, and 47
Herzog [10]	1987	7/F	84 and 85
Papageorge et al. [2]	1987	6/M	85
Cannell [11]	1991	8/F	85
Schafer et al. [3]	2001	8/F	85
Ohki et al. [12]	2004	12/M	85, 44, 45, 46, and 47
Lemberg et al. [13]	2007	11/F	85
Vieira et al. [14]	2007	7/F	75
Netto et al. [15]	2012	4/F	74
Monti et al. [16]	2013	11/F	75
Urs et al. [17]	2016	10/M	54, 55
Present case	2016	7/F	85
